# Advantages of 18F-FDG PET/CT Imaging over Modified Duke Criteria and Clinical Presumption in Patients with Challenging Suspicion of Infective Endocarditis

**DOI:** 10.3390/diagnostics11040720

**Published:** 2021-04-18

**Authors:** Valentin Pretet, Cyrille Blondet, Yvon Ruch, Matias Martinez, Soraya El Ghannudi, Olivier Morel, Yves Hansmann, Thomas H. Schindler, Alessio Imperiale

**Affiliations:** 1 Nuclear Medicine and Molecular Imaging, ICANS, University Hospitals of Strasbourg, rue Albert Calmette, 67093 Strasbourg, France; valentin.pretet@hotmail.com (V.P.); c.blondet@icans.eu (C.B.); matias.martinezag@hotmail.com (M.M.); soraya.elghannudi-abdo@chru-strasbourg.fr (S.E.G.); 2 Faculty of Medicine, FMTS, University of Strasbourg, 67000 Strasbourg, France; yvon.ruch@chru-strasbourg.fr (Y.R.); yves.hansmann@chru-strasbourg.fr (Y.H.); 3 Infectious Diseases, University Hospitals of Strasbourg, 67000 Strasbourg, France; 4 Nuclear Medicine and Molecular Imaging, Oulton Institute, 5000 Cordoba, Argentina; 5 Nuclear Medicine, Hospital Privado Universitario, 5000 Cordoba, Argentina; 6 Radiology, University Hospitals of Strasbourg, 67000 Strasbourg, France; 7 Cardiology, University Hospitals of Strasbourg, 67000 Strasbourg, France; olivier.morel@chru-strasbourg.fr; 8 Mallinckrodt Institute of Radiology, Division of Nuclear Medicine, Washington University, St Louis, MO 63110, USA; thscindler@wustl.edu; 9 Molecular Imaging–DRHIM, IPHC, UMR 7178, CNRS, 67037 Strasbourg, France

**Keywords:** infective endocarditis, modified Duke criteria, 18F-FDG PET/CT

## Abstract

According to European Society of Cardiology guidelines (ESC2015) for infective endocarditis (IE) management, modified Duke criteria (mDC) are implemented with a degree of clinical suspicion degree, leading to grades such as “possible” or “rejected” IE despite a persisting high level of clinical suspicion. Herein, we evaluate the ^18^F-FDG PET/CT diagnostic and therapeutic impact in IE suspicion, with emphasis on possible/rejected IE with a high clinical suspicion. Excluding cases of definite IE diagnosis, 53 patients who underwent ^18^F-FDG PET/CT for IE suspicion were selected and afterwards classified according to both mDC (possible IE/Duke 1, rejected IE/Duke 0) and clinical suspicion degree (high and low IE suspicion). The final status regarding IE diagnosis (gold standard) was based on the multidisciplinary decision of the Endocarditis Team, including the ‘imaging specialist’. PET/CT images of the cardiac area were qualitatively interpreted and the intensity of each focus of extra-physiologic ^18^F-FDG uptake was evaluated by a maximum standardized uptake value (SUVmax) measurement. Extra-cardiac ^18^F-FDG PET/CT pathological findings were considered to be a possible embolic event, a possible source of IE, or even a concomitant infection. Based on the Endocarditis Team consensus, final diagnosis of IE was retained in 19 (36%) patients and excluded in 34 (64%). With a sensitivity, specificity, positive predictive value (PPV), negative predictive value (NPV), and global accuracy of 79%, 100%, 100%, 89%, and 92%, respectively, PET/CT performed significantly better than mDC (*p =* 0.003), clinical suspicion degree (*p* = 0.001), and a combination of both (*p* = 0.001) for IE diagnosis. In 41 patients with possible/rejected IE but high clinical suspicion, sensitivity, specificity, PPV, NPV, and global accuracies were 78%, 100%, 100%, 85%, and 90%, respectively. Moreover, PET/CT contributed to patient management in 24 out of 53 (45%) cases. ^18^F-FDG PET/CT represents a valuable diagnostic tool that could be proposed for challenging IE cases with significant differences between mDC and clinical suspicion degree. ^18^F-FDG PET/CT allows a binary diagnosis (definite or rejected IE) by removing uncertain diagnostic situations, thus improving patient therapeutic management.

## 1. Introduction

Infective endocarditis (IE) is an uncommon and challenging infectious disease associated with severe complications and high mortality despite major advances in diagnostic and therapeutic strategies [1,2]. In the last few decades, we have witnessed a continuous evolution of the diagnostic criteria used in clinical practices for the management of patients with suspected IE. In 2000, modified Duke criteria (mDC), including clinical, echocardiographic, and biological findings, as well as blood cultures and serological results, were recommended for diagnostic use [3,4]. However, mDC show inadequate accuracy for IE diagnosis, particularly in patients with prosthetic valve endocarditis (PVE) and pacemaker or defibrillator lead IE, for which echocardiography is inconclusive in up to one-third of patients [5]. This is particularly true in early stages of the infectious process when the physician could have high clinical suspicion without a corresponding adequate mDC score for making the IE diagnosis. Consequently, the concept of clinical probability of IE (i.e., presumption of infection) has been merged with mDC in the recent diagnostic recommendations of the European Society of Cardiology (ESC) [4]. Hence, at present, diagnosis of IE is usually based on clinical probability in combination with mDC (definite, possible, or rejected IE), which are dependent on the diagnostic accuracy of either transthoracic (TTE) or transesophageal echocardiography (TEE). It is therefore possible to grade patients as having “possible” or even “rejected” IE despite a persisting high level of clinical suspicion [3,4].

As highlighted in 2015 by the revised guidelines of the European Society of Cardiology (ESC) [4], imaging is becoming increasingly significant for the management of patients with suspected IE [6]. Indeed, new techniques are becoming more frequently used when TTE/TEE results are negative or doubtful in both “possible” and “rejected” diagnoses, with a persistent high level of clinical suspicion. 18F-fluorodesoxyglucose (18F-FDG) positron emission tomography/computed tomography (PET/CT) emerged as a critical tool for identification of infected cardiovascular device implantations in cases where echocardiographic findings remain equivocal or negative despite a high clinical suspicion [7,8]. In addition, whole body 18F-FDG PET/CT potentially affords the identification of extra-cardiac primary infection sources and the extent of spread of the infection (infective embolism) [9].

Although the addition of a cardiac imaging exploration, be it MRI, CT, or PET/CT, is generally not recommended by ESC2015 guidelines, the interest of 18F-FDG PET/CT in patients with Duke 2 diagnosis (definite IE) has been shown by Duval et al. [10] in a recent prospective multicentric study, including 140 patients with IE suspicion and prosthetic (PV) or native valves (NV). When systematically performed, 18F-FDG PET/CT modified care in 31% of the 80 Duke 2 patients included in the study. Interestingly, Philip et al. [11] recently highlighted the central role of 18F-FDG PET/CT imaging in ESC2015 guidelines, resulting in correct patient reclassification from rejected or possible IE into definite IE. In their study, authors reported a 18F-FDG PET/CT sensitivity of 83.5%, including 53 out of 115 cases of definite IE.

To this day, few studies have specifically focused on patients presenting with discordance between mDC and clinical presumption of ongoing infection [7,8]. In view of the above, the aim of the present retrospective single-center study was to evaluate the diagnostic and therapeutic impact of 18F-FDG PET/CT in the management of challenging cases of IE suspicion, with emphasis on patients with possible/rejected IE but with high clinical suspicion.

## 2. Materials and Methods

### 2.1. Patient Population

This is a non-interventional, monocentric, retrospective study involving patients addressed to the Nuclear Medicine Department of the Strasbourg University Hospitals (France) for ^18^F-FDG PET/CT between May 2013 to November 2019 for suspected IE concerning the native valve or prosthetic material in the cardiac area (i.e., prosthetic valve, pacemaker lead, intra cardiac left ventricular assist device (LVAD), or other devices). Patients with a definite IE diagnosis, according to mDC, and patients with suspected infection developed on extra cardiac material (i.e., prosthetic vascular devices, pacemaker boxes, extra cardiac LVAD, and other devices) were not included in the analysis. 

**Figure 1:**
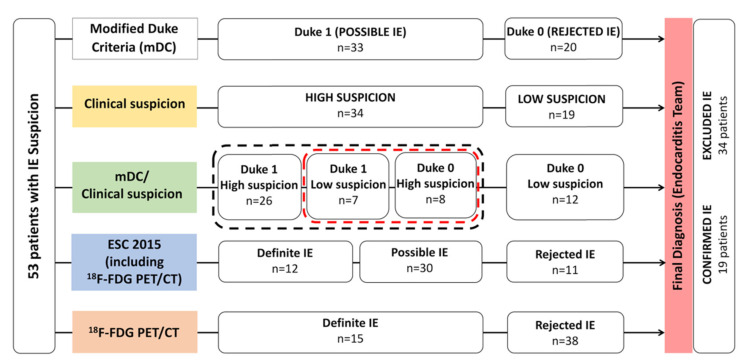
Main study design and patient population classifications. Black dotted line regroups patients needing cardiac imaging (Cardiac-CT, 18F-FDG PET/CT, or Cardiac-MRI) according to ESC 2015 guidelines. Red dotted line regroups the strongest classification discordances.

In the present study, each patient with IE suspicion has been classified twice, according to both mDC and clinical suspicion degree. Patients were initially categorized as possible IE (Duke 1) or rejected IE (Duke 0) and afterwards graded based on the clinical suspicion of infection (high or low suspicion of IE) (Figure 1). The clinical suspicion of endocarditis was defined by an infectious disease specialist based on available clinical, laboratory, and echocardiogram data. This was a subjective clinical judgment. Finally, patients were categorized considering both mDC (Duke 1 or 0) and clinical suspicion of IE (high or low suspicion). This approach was carried out blinded to the results of ^18^F-FDG PET/CT. This methodology is based on ESC2015 recommendations, which combine mDC (Duke 0 = rejected, 1 = possible, 2 = definite) and clinical suspicion of IE (low vs. high suspicion) in order to classify patients into three groups: definite IE, possible/rejected IE but high suspicion, and rejected IE with low suspicion. Indeed, even if the diagnostic probability of IE is rejected by mDC, the clinical presumption can be strong enough (i.e., high suspicion degree) to consider a rejected IE as a possible IE [4], justifying further imaging examinations.

### 2.2. ^18^F-FDG PET/CT: Technical Features and Interpretation Criteria

All ^18^F-FDG PET/CT were performed using an EARL-accredited combined PET/CT device (GE Healthcare, Chicago, IL, USA until April 2014, then Biograph mCT TOF, Siemens, Berlin, Germany) following the guidelines of the European Association of Nuclear Medicine (EANM). No patient with a previous history of thoracic surgery underwent ^18^F-FDG PET/CT in the first three postoperative months. PET/CT acquisitions started about 60 min after 3–5 MBq/kg of ^18^F-FDG injection (Flucis, IBA, Paris, France), including a head to midthigh non-contrast enhanced CT (128 detectors-row, 140 kV, 115 mA, 1s per rotation, pitch 0.8, slice thickness of 1 mm), followed by a PET scan (3–5 min/field). In order to minimize physiological myocardial ^18^F-FDG uptake, a high fat and low carbohydrate diet 24 h before the examination followed by 12 h of fasting was observed. Twenty-seven patients also received 10–20 UI/kg standard heparin intravenous administration about 30 min before ^18^F-FDG injection. ECG-gated acquisition or contrast-enhanced PET/CTA was not performed in any case. PET data were reconstructed with and without CT-based attenuation correction using the common iterative algorithm OSEM. CT, PET, and combined PET/CT images were displayed on a dedicated workstation (Syngo.via VB30, Siemens, Berlin, Germany) and analyzed by two experienced nuclear medicine physicians. In case of disagreement, a third nuclear physician was required to reach consensus.

Attenuation-corrected and non-attenuation-corrected PET/CT images of the cardiac area were qualitatively interpreted according to widely accepted criteria (pronounced focal and/or heterogenous vs. absence of or homogenous mild ^18^F-FDG uptake around the device) [12,13]. Although in clinical practice the use of standardized uptake value (SUV) is debated and not always recommended [14,15], we have assessed SUVmax of each focus of extra-physiologic uptake of ^18^F-FDG in cardiac area. The patient’s clinical situation and previous history were considered for the assessment and interpretation of increased extra-physiologic uptake of ^18^F-FDG. Extra-cardiac ^18^F-FDG PET/CT pathological findings were considered to be a possible embolic event, a possible source of IE, or even a concomitant infection. ^18^F-FDG was used in the setting of an approved marketing authorization and a cross-disciplinary team, including clinicians and nuclear medicine physicians who stated about ^18^F-FDG PET/CT indications. In accordance with local institutional guidelines, all patients included gave free and informed consent for the use of anonymous personal medical data extracted from their file for scientific or epidemiological purposes. 

### 2.3. Statistical Analysis

Results for continuous data were expressed as mean ± standard deviation or median and range as appropriate. Categorical variables were presented as numbers and percentages. The Wilcoxon–Mann–Whitney *U* test and the McNemar test were used for comparison. ^18^F-FDG PET/CT sensitivity (Se), specificity (Sp), positive predictive value (PPV), negative predictive value (NPV), and global accuracy (Ac) were calculated for each patient. The final status regarding IE diagnosis (gold standard) was based on the multidisciplinary decision of the Endocarditis Team, including the ‘imaging specialist’ [16]. Two-sided *p* values < 0.05 were considered significant. Statistical analyses were performed using an open access statistical software (biostatgv.sentiweb.fr, 2020).

## 3. Results

### 3.1. Overall Population

Fifty-three patients with suspected IE were enrolled during the study period. Table 1 summarizes the characteristics of patient population. Mean age was 65 ± 19 years with a male to female ratio of 3:2. In total, 43 out of 53 (81%) patients had a cardiac device, 22 (41%) a prosthetic valve, 24 (45%) a cardiac electronic implantable device (CEID), 4 (8%) a LVAD, and other devices were in the last 4 cases. Five (9%) of the patients had at least two prosthetic valves and nine (17%) had at least one prosthetic valve and a CEID (Table 1). Blood culture revealed causative pathogen in 24 out of 53 included patients (45%). In the overall population, TTE and TEE were positive in 6 (12%) and 13 (30%) patients, respectively. According to mDC criteria, 20 (38%) patients were classified as Duke 0 and 33 (62%) as Duke 1. According to the Infectious Disease Specialist Consensus, 34 (64%) and 19 (36%) patients were classified with having low or high clinical suspicion of IE, respectively. Merging both mDC and clinical suspicion, 26 (49%) patients were classified as having Duke 1/high suspicion, 7 (13%) patients as having Duke 1/low suspicion, 8 (15%) patients as having Duke 0/high suspicion, and 12 (23%) patients as having Duke 0/low suspicion (Figure 1). Finally, based on the Endocarditis Team decision [16], the final diagnosis of IE was retained in 19 (36%) patients and excluded in the remaining 34 (64%). IE occurred on the prosthetic mitral valve in 2 (11%) cases, on the prosthetic aortic valve in 3 (16%), on the prosthetic pulmonary valve in 2 (11%), on the native mitral valve in 2 (11%), on the CEID intra cardiac lead in 6 (32%), on the surgical patch closure of the atrial septum defect in 1 (5%), and on the LVAD in 3 (16%). Blood culture were conclusive in 12 out of 19 cases (63%) with final diagnosis of EI and in 12 of 34 patients (35%) without final diagnosis of EI. The main isolated pathogens responsible for infection in patients with EI final diagnosis were Staphylococcus aureus (*n* = 4), Coagulase Negative Staphylococcus (*n* = 3), Enterococcus spp. (*n* = 3), and Streptococcus spp. (*n* = 2). In 19 patients with final diagnosis of EI, TTE and TEE were positive in 3 (16%) and 4 (24%) cases, respectively. 

### 3.2. Modified Duke Criteria (mDC), Clinical Suspicion Degree, and Combined mDC/Clinical Suspicion

To assess the diagnostic performances of mDC, Duke 1 patients were considered to have a positive IE diagnosis and Duke 0 a negative IE diagnosis. In the same manner, patients with high and low suspicion were considered to have a positive and negative IE diagnosis, respectively. Combining both mDC and clinical suspicion degree, positive diagnosis of IE was retained for patients presenting with Duke 1/high suspicion, Duke 1/low suspicion, and Duke 0/high suspicion [4]. On the other hand, patients with Duke 0/low suspicion were considered to have a negative IE diagnosis [4]. Thus, according to the final diagnosis assessed by the institutional multidisciplinary Endocarditis Team, the Se, Sp, PPV, NPV, and Ac were, respectively, 84%, 50%, 48%, 85%, and 62% for mDC and 95%, 53%, 53%, 95%, and 68% for clinical suspicion degree. Taking into account both mDC and the clinical suspicion degree, the Se, Sp, PPV, NPV, and Ac were 95%, 32%, 44%, 92%, and 55%, respectively (Table 2). A head-to-head comparison of global accuracy for IE diagnosis between mDC, clinical suspicion degree, and the combination of both showed no statistically significant difference (Table 3).

Using mDC, 3/20 (15%) Duke 0 patients had a final diagnosis of IE, while the Endocarditis Team excluded IE diagnosis in 17 out of 33 (52%) Duke 1 patients. According to clinical suspicion degree, 1 out of 19 (5%) low suspicion patients had a final diagnosis of IE. On the other hand, in 16 out of 34 (47%) high suspicion patients, IE diagnosis was finally excluded. Merging mDC and clinical suspicion degree, the diagnosis of IE was retained in 17 out of 26 (65%) Duke 1/high suspicion patients, 2 out of 8 (25%) Duke 0/high suspicion patients, and 1 out of 12 (8%) Duke 0/low suspicion patients. Finally, the Endocarditis Team concluded an absence of IE in 7 out of 7 Duke 1/low suspicion patients.

### 3.3. ^18^F-FDG PET/CT

#### 3.3.1. Cardiac Area Investigation

**Figure 2:**
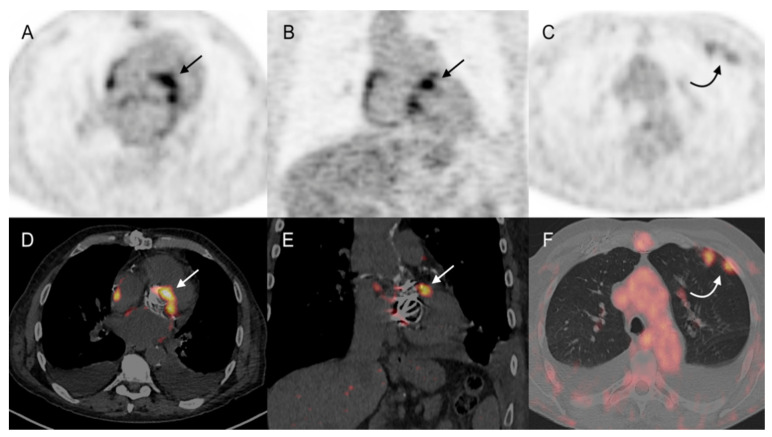
73-year-old man with aortic and mitral mechanic valve prostheses presented with fever and blood cultures positive for Streptococcus gallolyticus (Duke 1/high clinical suspicion degree). TTE and TEE were both negative. PET/CT showed increased focal ^18^F-FDG uptake between the aortic and mitral mechanic valves (arrows) and in left lung parenchymal condensations (curved arrows). According to the Endocarditis Team, final diagnosis was an infected mechanic aortic valve with pulmonary septic emboli. (**A**–**C**): attenuation-corrected PET, axial, and coronal slices; (**D**–**F**): attenuation-corrected PET/CT, axial, and coronal slices.

**Figure 3:**
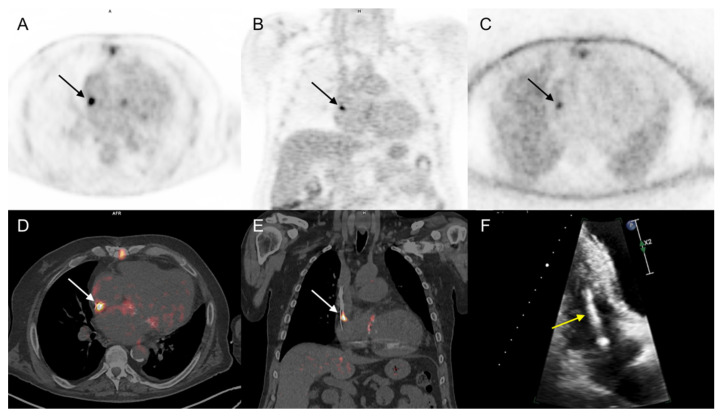
68-year-old man with a history of implantable cardioverter defibrillator (ICD) and a biological prosthetic mitral valve presented with 10-mm enlargement of ICD lead within the right auricle at TTE (yellow arrow) but normal routine biological evaluation and negative blood cultures (Duke 1/high clinical suspicion degree). PET/CT showed increased focal ^18^F-FDG uptake, corresponding to right auricle ICD lead (arrows). The Endocarditis Team’s final diagnosis was CIED IE, and ICD was removed. (**A**,**B**): PET, attenuation-corrected axial, and coronal slices. (**C**): PET, non-attenuation corrected axial slice. (**D**,**E**): PET/CT, axial, and coronal attenuation corrected slices. (**F**): TEE.

Forty (75%) patients received wide spectrum antibiotic treatment at the time of the 18F-FDG PET/CT examination. In a per patient analysis (PET/CT positive or negative for IE on cardiac area), two interpreting nuclear medicine physicians were concordant for 48 of the studied 53 patients. A third physician was required for reaching a consensus in the remaining 5 patients. In the whole population, 18F-FDG PET/CT Se, Sp, PPV, NPV, and Ac for IE diagnosis were 79%, 100%, 100%, 89%, and 92%, respectively. Considering only the 43 patients with prosthetic valves or any other cardiac devices, 18F-FDG PET/CT sensitivity was slightly better (83%) than that reported from the analysis of the whole population (Table 2). 18F-FDG PET/CT was considered a true positive for the presence of IE in 15 out of 19 patients with final diagnosis of IE (Figure 2 and Figure 3).

In the remaining four patients, 18F-FDG PET/CT failed to detect the native mitral valve IE (*n* = 2), prosthetic mitral valve IE (*n* = 1), and CEID lead IE (*n* = 1). False negative results were mainly due to an insufficient decrease of physiological 18F-FDG myocardial uptake following the high fat diet (17–19). Moreover, all four patients with false negative results were treated by large spectrum antibiotics at the time of PET/CT. No false positive ^18^F-FDG PET/CT study was registered. 

Patients with a final diagnosis of IE showed higher SUVmax values assessed on cardiac area abnormalities than no-IE patients (SUVmax: 6.2 vs. 4.9, *p* = 0.05). However, the small sample analyzed did not allow the definition of a SUVmax diagnostic threshold for a reliable clinical use. Overall, 18F-FDG PET/CT performed significantly better than mDC (*p* = 0.003), clinical suspicion degree (*p* = 0.001), and the combination of both (*p* = 0.001) for IE diagnosis (Table 3). When 41 patients with possible or rejected IE but high clinical suspicion were analyzed jointly (4), 18F-FDG PET/CT revealed a Se, Sp, PPV, NPV, and Ac of, respectively, 78%, 100%, 100%, 85%, and 90%.

In the subgroup of 15 patients with the strongest classification discordances, including only Duke 0/high suspicion (*n* = 8) and Duke 1/low suspicion (*n* = 7), ^18^F FDG PET/CT correctly identified all patients (Table 4). When the results of ^18^F-FDG PET/CT were considered as a major criterion within ESC2015 guidelines, 1 out of 12 (8%) rejected IE diagnoses (Duke 0/low suspicion) was changed to a possible IE diagnosis, and 11 out of 41 (27%) possible/rejected IE diagnoses with high clinical suspicion were changed to definite IE diagnoses.

#### 3.3.2. Extra-Cardiac Infection Assessment

In 9 out of 19 patients with a final diagnosis of IE, PET/CT showed extra-cardiac 18F-FDG pathologic uptake in: (a) lung (*n* = 3) and bone (*n* = 1), suggesting embolic spread (Figure 2); (b) previously documented spondylodiscitis (*n* = 1) and infected hip and knee prosthesis (*n* = 2), which were afterwards identified as the source of IE; (c) parietal collections (*n* = 2) corresponding to post-thoracotomy abscesses, which were finally considered as concomitant infections independent from IE. Concerning the 34 patients with excluded IE diagnosis, 18F-FDG PET/CT showed pathologic extra-cardiac uptake in 17 cases, strongly suggesting lung infections (*n* = 6), spondylodiscitis (*n* = 3), post-thoracotomy local abscess (*n* = 1), infected knee prosthesis (*n* = 1), infected central catheters (*n* = 2), colitis (*n* = 1), infected deep venous thrombosis (*n* = 1), LVAD cable infection in the abdominal path (*n* = 1), and infected acute pancreatitis associated with cholecystitis (*n* = 1) when correlated with patient follow up. In 6 out of 12 patients with initially rejected (Duke 0/Low suspicion) and finally excluded IE diagnoses, 18F-FDG PET/CT contributed to detected lung infections (*n* = 3), infected acute pancreatitis associated with cholecystitis (*n* = 1), infected central catheter (*n* = 1), and infection of the extra thoracic cable of a LVAD (*n* = 1).

#### 3.3.3. Impact on Patient Management

The real therapeutic impact of 18F-FDG PET/CT in a retrospective setting could be difficult to assess. Patient therapeutic strategy was led by the Endocarditis team. Accordingly, 18F-FDG PET/CT contributed to patient management in 24 out of 53 (45%) cases. In particular, PET/CT was considered contributory when it fulfilled at least one of the following criteria: (a) identification of the presence of cardiac infection (other conventional tests performed at the time of PET/CT imaging were negative and/or non-contributory) (*n* = 15, 28%), (b) determination of the extent of the infection or the involvement of other organs (*n* = 4, 8%), (c) removal of the infection site (*n* = 3, 6%, including 2 CEID removals and 1 PV replacement), and (d) change in the nature and duration of antibiotic therapy (*n* = 14, 26%): beginning of specific antibiotic treatment (*n* = 2) and modification of antibiotic spectrum (*n* = 12).

## 4. Discussion

The main findings that can be drawn from our retrospective study are in line with ESC2015 recommendations, highlighting the good performance of ^18^F-FDG PET/CT for the diagnosis of IE, doing better than the mDC, the degree of clinical suspicion, and the combination of both [17,18,19,20,21,22,23]. ^18^F-FDG PET/CT sensitivity for IE detection was higher in patients with cardiac devices than in the overall population (83% vs. 79%, respectively). Moreover, ^18^F-FDG PET/CT was efficient for 41 patients with possible/rejected IE but high clinical suspicion (Se: 78%), a situation which seems to represent the most difficult cases [4]. Finally, ^18^F-FDG PET/CT correctly characterized all 15 patients classified as Duke 0/high suspicion and Duke 1/low suspicion.

The clinical interest of ^18^F-FDG PET/CT in patients with possible/rejected IE but high clinical suspicion or rejected IE and low clinical suspicion needs further evaluation. Therefore, we have challenged ^18^F-FDG PET/CT by including only patients without a definite diagnosis of IE, according to mDC (i.e., only Duke 0 and 1). Consequently, patients presented with elevated inflammatory blood markers or at least one minor criterion according to mDC (Duke 0 and low clinical suspicion) were included in the analysis. ^18^F-FDG PET/CT for these patients were largely performed before publication of the ESC2015 guidelines. Finally, a sensitivity of 78% in patients with possible or rejected IE but high clinical suspicion appears robust. Moreover, ^18^F-FDG PET/CT plays a role in patient management in 24 out of 53 (45%) cases, in accordance with previously reported data [9,10,24].

Despite disagreements in the literature, there is a growing interest for early ^18^F-FDG PET/CT use in the diagnostic algorithm in IE suspicion [10,11]. Moreover, early ^18^F-FDG PET/CT for IE suspicion seems to lead to a prompt treatment and a better prognosis [25]. The inclusion of ^18^F-FDG PET/CT in the ESC2015 guidelines led to a 3-scale degree of IE probability, resulting in numerous doubtful diagnostic situations. In our study, when ^18^F-FDG PET/CT was considered as a major criterion for IE diagnosis in the ESC2015 guidelines, 12 and 11 patients were respectively reclassified from possible into definite and rejected IE. However, the diagnosis of possible IE persisted as unchanged for the remaining 30 patients (57%). According to our findings, ^18^F-FDG PET/CT defines the diagnosis as either rejected or definite IE. Thus, adding ^18^F-FDG PET/CT directly to mDC at patient admission could promptly reduce the number of possible and rejected IE by increasing specificity, without a major change of sensitivity.

In our series, ^18^F-FDG PET/CT failed to detect native mitral valve IE in two patients. According to recent data, PET cardiac studies should be performed as ECG-gated and contrast-enhanced PET/CTA to increase the diagnostic accuracy, particularly for the evaluation of native valves [16,26,27,28]. However, both techniques are not widely available in all nuclear medicine laboratories, and the present study could be considered as an illustration of “real life” ^18^F-FDG PET/CT utilization. In our series, ^18^F-FDG PET/CT showed optimal specificity without achieving false positive results and good diagnostic accuracy, despite the absence of ECG-gated PET acquisition and contrast media injection. These findings are the consequences of an expert qualitative analysis using well-defined interpretation criteria, as previously mentioned [12,13,29].

CIED represents a specific diagnostic and therapeutic challenge. In our study, 18F-FDG PET/CT failed to detect one patient with CEID-lead IE. Intra-cardiac echocardiography (ICE) is a diagnostic alternative, without patient radiation exposure, useful in high clinical suspicion of CIED when TTE and TEE are non-conclusive. However, it rests on an invasive diagnostic technique [30,31]. 

Semi-quantitative analysis using standardized uptake (SUV) has been proposed but not validated in inflammation and infection. Several authors previously reported conflicting results without reaching a definitive threshold that would allow to distinguish between possible and rejected IE diagnosis in daily clinical practices [14,15]. The additional value of quantitative parameters, such as SUVmax, in differentiating between infected and noninfected material is still debated. New semiquantitative indices have recently been examined but need further confirmation [32].

Four patients with a final diagnosis of IE reported a false negative result from their PET/CT investigation. All of these patients were being treated by large spectrum antibiotics at the time of the PET/CT examination. Chronic or indolent infections with slow-growing bacteria, or infections with bacteria in a quiescent status after long-term antibiotic treatments, are common causes of false negative ^18^F-FDG PET/CT results. Moreover, in clinical practice, most patients with suspicion of IE generally have started antibiotic therapy before the PET/CT examination. Therefore, it is advisable to conduct a ^18^F-FDG PET/CT study before treatment initiation or as quickly as possible to avoid potential underestimation of disease extent and severity. If there is clinical suspicion of infection, a negative result authorizes clinical observation without immediate surgery, while a positive ^18^F-FDG PET/CT finding in the clinical context may lead to the extraction of the entire device. Thus, it is important to bear in mind that a false-negative PET result, which is often related to a mild or chronic infection after or during the antibiotic treatment, may not really influence conservative management and has a low risk of exposing the patient to clinical deterioration.

Recent studies show that early identification of embolic events or infection sources using ^18^F-FDG PET/CT means early antimicrobial therapy (adapted to a suspected causing pathogen according to the infectious gateway), prolonged or modified treatment, or even early surgical cardiac valve procedures [33,34]. In our study, ^18^F-FDG PET/CT suggested extra-cardiac infectious processes in 6 out of 12 patients with a final excluded IE. Concerning the 19 patients with a confirmed IE, ^18^F-FDG PET/CT revealed extra-cardiac pathological uptake in 9 out of 19 (47%) of them, which was determined to be related to either septic embolic events, sources of the infection, or concomitant infection. These data match the performances of ^18^F-FDG PET/CT in the assessment of unexplained chronic fever or inflammatory syndrome [35].

The use of multimodality imaging could be of great interest for the management of IE patients [36]. Functional techniques will be variably associated with morphological investigations. A typical example of diagnostic synergy is the combined use of ^18^F-FDG PET and CTA in a single PET/CTA examination [15]. Nowadays, PET/MRI hybrid systems decrease the effective radiation doses and are beginning to be clinically available. However, further studies with long-term economic considerations will be needed to validate and support the necessity for this imaging approach. The optimal combination of imaging techniques necessitates determination to improve the diagnostic accuracy and reduce non-essential patient radiation exposure [37,38].

This study presents several limitations that deserve comments: first, its retrospective and monocentric design focused on a heterogeneous population, including cases of PVE, devices, and LVAD infection. However, it could be considered as a basis for future investigational prospective multicenter studies, including a larger number of patients with challenging endocarditis on native valves and devices, proving that ^18^F-FDG PET/CT is feasible in most centers. Second, the number of included patients is also quite small compared to recent studies on the same topic, which is mainly related to different inclusion criteria. Indeed, in our work, patients with a definite diagnosis of IE were not included, contrarily to other recent studies based on larger patient cohorts. Next, the clinical suspicion degree of IE diagnosis was established by the Infectious Disease Specialist Consensus, which could be based on relatively subjective criteria but does represent a picture of real-life clinical practices. Finally, as previously mentioned, some patients had ^18^F-FDG PET/CT exploration before publication of the ESC guidelines, potentially representing a patient selection bias.

## 5. Conclusions

^18^F-FDG PET/CT represents a valuable diagnostic tool that should be considered in patients with IE suspicion, even for those challenging cases with significant inconsistencies between mDC and clinical IE presumption. In this respect, ^18^F-FDG PET/CT may afford a binary diagnosis (definite or rejected IE) by removing uncertain diagnostic situations, thus improving patient therapeutic management. 

## Figures and Tables

**Table 1:** Population main characteristics.

Characteristics	Values
Age (years), mean ± SD	65 ± 19
Sex, *n* (%)	
Female Male	20 (38) 33 (62)
CRP (mg/L), mean (range)	81.9 (4.0–280 ; N<4)
White blood cell (G/L), mean (range)	10.0 (2.8–20.0 ; 4.1<N<10.5)
Material, *n* (%) Prosthetic valve * Biological Aortic Mitral Pulmonary Mechanic Aortic Mitral CEID LVAD Others **	43 (81) 22 (42) 15 (28) 11 (21) 5 (10) 2 (4) 9 (17) 4 (8) 6 (11) 24 (45) 4 (8) 4 (8)
Causative pathogen, *n* (%)	
Positive blood culture for IE Coagulase Negative *Staphylococcus* *Staphylococcus aureus* *Streptococcus* spp. *Enterococcus* spp.	12 (23) 3 (25) 4 (33) 2 (17) 3 (25)
Ongoing antibiotic treatment, *n* (%)	40 (75)
Modified Duke Criteria, *n* (%)	
Duke 0 (Rejected IE) Duke 1 (Possible IE)	20 (38) 33 (62)
Clinical suspicion, *n* (%)	
Low High	19 (36) 34 (64)
IE diagnostic probability, *n* (%)	
Duke 0/Low suspicion Duke 1/High suspicion Duke 1/Low suspicion Duke 0/High suspicion	12 (23) 26 (49) 7 (13) 8 (15)
Extra-cardiac FDG PET/CT infected site, *n* (%)	26 (49)

*: Two patients had both mitral and aortic biological valves, one patient had a mitral mechanical valve with an aortic biological valve, one patient had a pulmonary biological valve and an aortic mechanical valve, and one patient had both mitral and aortic mechanical valves; **: Two valve-tube grafts, one surgical patch closure of atrial septum defect. N: normal value.

**Table 2:** Overall diagnostic results compared to the final diagnosis, according to the Endocarditis Team consensus.

	Se	Sp	PPV	NPV	Ac
**Modified Duke Criteria (mDC)**	84% (16/19)	50% (17/34)	48% (16/33)	85% (17/20)	62% (33/53)
**Degree of Clinical Suspicion**	95% (18/19)	53% (18/34)	53% (18/34)	95% (18/19)	68% (36/53)
**mDC+Clinical Suspicion**	95% (18/19)	32% (11/34)	44% (18/41)	92% (11/12)	55% (29/53)
**^18^F-FDG PET/CT**	79% (15/19)	100% (34/34)	100% (15/15)	89% (34/38)	92% (49/53)
^18^F-FDG PET/CT *	83% (15/18)	100% (25/25)	100% (15/15)	89% (25/28)	93% (40/43)

*: 43 patients with prosthetic material.

**Table 3:** Head-to-head comparison (global accuracy for IE diagnosis) between mDC, clinical suspicion degree, the combination of both, and ^18^F-FDG PET/CT.

	Duke Modified Criteria (mDC)	Clinical Suspicion	mDC+Clinical SUSPICION Degree	^18^F-FDG PET/CT
**Modified Duke Criteria (mDC)**	-	ns	ns	*p =* 0.003
**Clinical Suspicion degree**	ns	-	ns	*p =* 0.001
**mDC+Clinical Suspicion degree**	ns	ns	-	*p* = 0.001
**^18^F-FDG PET/CT**	*p* = 0.003	*p* = 0.001	*p* = 0.001	-

**Table 4:** Fifteen patients with the strongest classification discordances, including Duke 0/high suspicion (*n* = 8) and Duke 1/low suspicion (*n* = 7). mDc: modified Duke Criteria; IE: infective endocarditis; Rej: mDC rejected IE; Poss: mDC possible IE; PM: pace-maker; LVAD: left ventricular assistance device; TTE: transthoracic echography; TEE: transesophagus echography; Ao veg: aortic valve vegetation; Mit veg: Mitral valve vegetation; Mit abs: mitral abscess.

*n*°, Age, Sex	mDC/Clinical Suspicion	Final Diagnosis	FDG PET	TTE	TEE	mDC Major Microbiological Evidence	mDC Minor Findings
1, 68, F	Rej/High	no IE	-	-	-	-	Predisposition, microbiologic evidence (*Pseudomonas aeruginosa*)
2, 43, M	Rej/High	no IE	-	-	-	-	vascular, phenomena
3, 78, M	Rej/High	no IE	-	-	-	-	predisposition, fever
4, 48, F	Rej/High	IE	PM lead	-	-	-	Predisposition, fever
5, 50, M	Rej/High	IE	LVAD	-	-	-	Fever, microbiologic evidence (*Staphylococcus aureus*)
6, 72, M	Rej/High	no IE	-	-	-	-	fever
7, 76, F	Rej/High	no IE	-	-	-	*Staphylococcus epidermidis*	-
8, 56, M	Rej/High	no IE	-	-	-	*Coxiella burnetii* IgG antiphase 1 > 1/800	-
9, 79, F	Poss/Low	no IE	-	-	-	*Staphylococcus aureus*	fever
10, 82, M	Poss/Low	no IE	-	-	-	*Staphylococcus epidermidis*	fever
11, 53, M	Poss/Low	no IE	-	Ao veg	Ao veg	-	predisposition
12, 31, F	Poss/Low	no IE	-	-	-	-	Predisposition, fever, microbiologic evidence (*Pantoea ananatis*)
13, 75, F	Poss/Low	no IE	-	Mit veg	Mit veg	-	fever
14, 81, M	Poss/Low	no IE	-	-	Mit abs	-	fever
15, 67, M	Poss/Low	no IE	-	-	-	*Staphylococcus epidermidis*	Predisposition, fever

## Data Availability

Purely observational studies do not require registration.
